# Supplementary material for: Synergistic pretraining of parametrized quantum circuits via tensor networks
Source: Nat Commun. 2023 Dec 15;14:8367. doi: 10.1038/s41467-023-43908-6 (PMC10724286; doi:10.1038/s41467-023-43908-6)
Supplement: Supplementary file 1 — Supplementary Information [file 41467_2023_43908_MOESM1_ESM.pdf]

Supplementary Information for
"Synergistic Pretraining of Parametrized Quantum Circuits via Tensor Networks"

Manuel S. Rudolph,[1] Jacob Miller,[1] Danial Motlagh,[1] Jing Chen,[2] Atithi Acharya,[2,3] and Alejandro Perdomo-Ortiz[1,*]

[1]*Zapata Computing Canada Inc., 325 Front St W, Toronto, ON, M5V 2Y1*
[2]*Zapata Computing Inc., 100 Federal Street, Boston, MA 02110, USA*
[3]*Rutgers University, 136 Frelinghuysen Rd, Piscataway, NJ 08854, USA*

Supplementary Note 1. DISTRIBUTION OF GRADIENT MAGNITUDES

To supplement the expected gradient variance statistics depicted in the main text, we now present a more fine-grained study of the gradients within individual models. For this, we pick the 16-qubit instance of the Cardinality dataset and calculate the entire gradient vector of four QCBMs with respect to the KL divergence loss function. The circuits are either 15 linear layers, or one all-to-all layer of $\mathrm{SU}(4)$ gates. We then record the distribution of gradient vector entries at random parameters or pretrained parameters from a $\chi = 2$ bond dimension TNBM. The results are shown in Fig. 1.

In the case of random initial parameters, it can be seen that the distribution of gradient magnitudes decays exponentially, making large gradients exponentially unlikely within one model. This is a pattern that is expected for exponential concentration phenomena in general, and is a form of *probabilistic* exponential concentration [1]. In contrast, the pretrained QCBMs completely break the trend observed in the random circuits and exhibit generous gradient magnitudes for a significant proportion of their parameters. By highlighting in red the pretrained parameters in Fig. 1, we determine that there is no clear trend which type of parameters (i.e., pretrained or near-identity initialized) tend to have larger gradients. This speaks to the positive influence of both the pretrained parameters and the additional gates. One may have expected that the pretrained parameters are in a local minimum, or more precisely, a saddle point. This would lead to systematically lower gradient for those parameters. At the same time, we observe that the newly added parameters experience large gradients, highlighting that these parameters will be able to be trained in order to improve the pretrained circuit. We attribute much of this positive behavior to the near-identity initialized with parameters that are drawn from a Gaussian distribution with zero mean and a standard deviation of 0.01.

Finally, we note that the variation in gradient distributions is quite large between individual pretrained models, which is consistent with the sizeable expected gradient variance displayed in Fig. 3 of the main text. The results shown here should therefore merely be understood as representative examples. The exponential decay in gradient magnitudes does however appear to be very robust for the randomly initialized QCBM at its respective number of qubits.

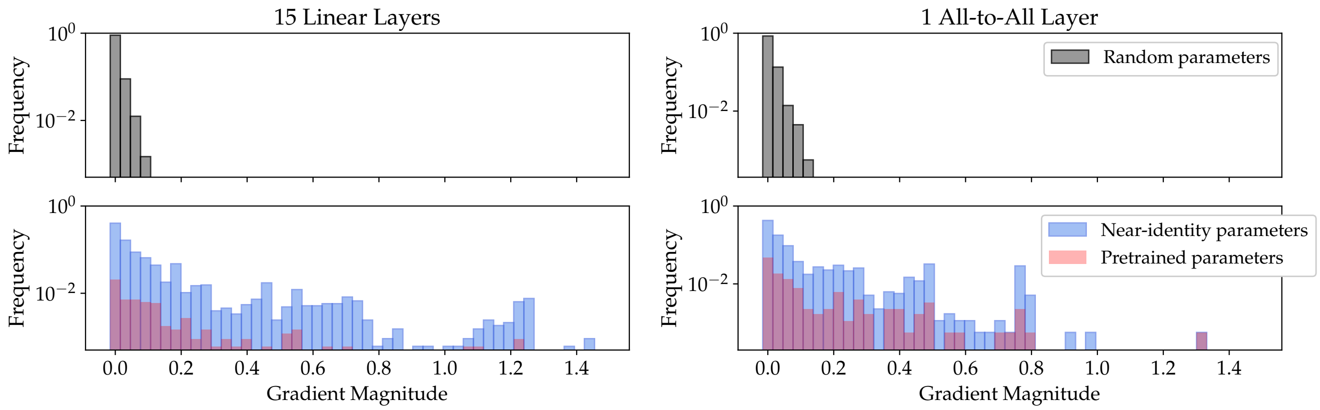

Supplementary Figure 1. Distribution of gradient magnitudes in the gradient vectors of individual 16-qubit QCBMs with random parameters or pretrained parameters using a $\chi = 2$ TNBM. The circuits consist of 15 linear layers (left) or one all-to-all layer (right) of $\mathrm{SU}(4)$ gates, where only the linear component of the first layer was pretrained.

* aperdomo@post.harvard.edu

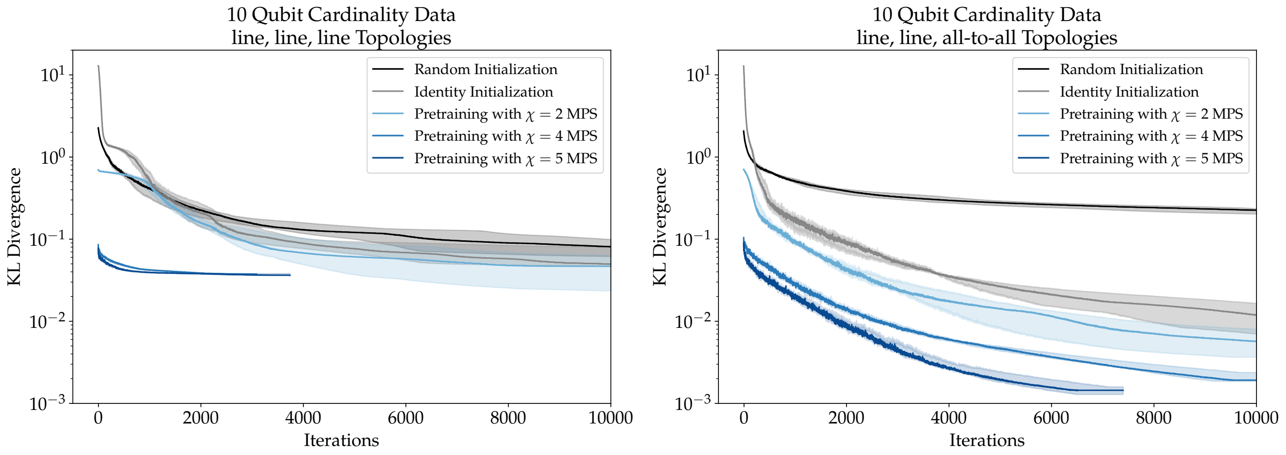

Supplementary Figure 2. Optimization results for QCBMs training on the Cardinality dataset with $N = 10$ qubits and three linear layers. We showcase the enhancement of the mapped MPS solutions when the final linear layer is extended to an all-to-all topology. While trainability suffers for random initial parameters, the classically initialized PQCs are able to converge to significantly better solutions.

Supplementary Note 2. PERFORMANCE ENHANCEMENT WHEN EXTENDING LINEAR LAYERS

When initializing PQCs for model optimization, the approach throughout this work has been to decompose the classically trained MPS into k linear layers of two-qubit gates, and extend only the final layer into an all-to-all topology. This is to say that we add additional two-qubit gates with near-zero parameters to the final linear layer until every qubit is directly connected to every other qubit. The two-qubit gates used are described in Eq. (1). The precise placement of additional gates here is meant to be generic, and the optimal and most resource-efficient method is likely highly problem-dependent. Fig. 2 of the main text demonstrates how PQCs with added gates can reliably improve on the final loss values that the MPS could achieve. In contrast, Fig. 2 depicts an example case of an $N = 10$ qubit QCBM training on the Cardinality dataset where we do not add these additional gates to the linear layers after transferring the MPS solution to the quantum circuit. Interestingly, with random initial parameters, the linearly arranged circuit reaches better KL divergence values (black, left panel) than when the final layer is extended to an all-to-all topology (black, right panel). This highlights the deterioration of trainability with increasing circuit depth that can also seen in Fig. 3 of the main text. The near-identity initialization is notably less hampered by this. When classically initializing the QCBMs, the models can only aim to recover the MPS performance when the circuit is not extended with additional gates and free parameters. In contrast, the all-to-all topology allows the QCBM to achieve great performance and, as also seen in Fig. 2 of the main text, QCBM initialized with larger χ MPS perform better even when starting at similar loss values.

Supplementary Note 3. MEDIAN OPTIMIZATION PERFORMANCE

In addition to the best of 6 optimization runs shown in Fig. 2 of the main text, we here report the median and 25-75 percentile of the losses throughout the optimization. The statistics were gathered by bootstrapping, which is a common data re-sampling strategy to enhance the robustness of uncertainty estimates. Fig. 3 shows that initializing the PQC circuits with classically found MPS solutions is beneficial in all cases and significantly enhances the trainability of the models. In the cases of the Cardinality dataset in the generative modeling task, and the Heisenberg ground state minimization, the synergistic optimization framework shows continuous improvements with increasing MPS bond dimension χ. The case of the BAS dataset is different. As discussed in main text, the BAS dataset is a dataset which exhibits strong correlations between bits within the same row or column, which means that it is a 2d-correlated dataset. On the other hand, the MPS solutions, due to the line-graph of the TN, are particularly well-suited to represent 1d-correlated states. This introduces an unbeneficial bias miss-match which the PQC after circuit extension needs to un-learn. In such cases, initializing the PQC with large χ MPS may not deliver the desired robust improvements. Still, the synergistic approach with any χ greatly outperforms the uninformed random initialization and also the near-identity initialization, which was empirically shown to deal with barren plateaus [2].

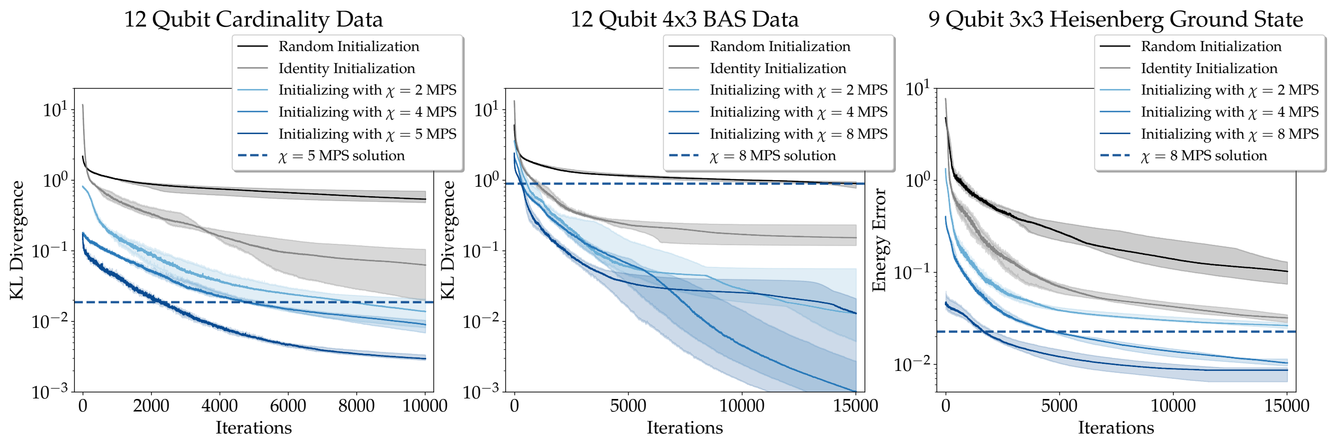

Supplementary Figure 3. Optimization results for QCBM and VQE training, where each curve represents the bootstrapped median and 25-75 percentiles of 6 independently initialized runs of the model. The data is complementary to the best of 6 runs shown in Fig. 2 of the main text. Initializing the PQCs with classically found MPS solutions appears to be generally beneficial as compared to randomly or near-identity initialized parameters. While continuous improvements can be attained with larger bond dimension χ in the Cardinality dataset, as well as the Heisenberg ground state minimization, the BAS dataset - which is a 2D correlated dataset - exhibits an unfavorable bias miss-match with the MPS that the PQC needs to un-learn. Even so, the synergistic framework with small χ greatly improves on the uninformed initializations.

Supplementary Note 4. IMPLEMENTATION DETAILS OF THE MPS AND PQCS

A. MPS optimization

The MPS for the generative modelling task, i.e., the TNBMs, were implemented and trained according to the method outlined in Ref. [3]. The TNBMs are trained via gradient descent using gradients of the KL divergence loss function that can be calculated efficiently using a *density matrix renormalization group* (DMRG) [4] method. This approach allows for adaptive bond dimensions χ for each MPS bond up to a maximum of χ_{max}, or a singular value threshold of $5 \cdot 10^{-5}$ in the SVD truncation. The learning rate η for the gradient descent updates was chosen to be $\eta = 0.01$, with 50 optimization sweeps for the results in Fig. 2 of the main text for complete convergence, and 30 optimization sweeps for the gradient results in Fig. 3 of the main text.

The training of the MPS for Hamiltonian ground state minimization uses an analogous DMRG-based gradient calculation approach for the energy loss function described in the main text. The MPS calculations utilize the ITensor library [5], were $\chi = 16$ is used to find the exact ground state without any approximation. The exact MPS was then truncated via SVD to $\chi = 2, 4, 8$ for the results in Fig. 2 of the main text. This is viable because fidelity with the ground state (which is what SVD empirically optimizes) and the energy are proportional, whereas fidelity is not proportional to the KL divergence.

B. SU(4) gates

The PQCs throughout this work used $\mathrm{SU}(4)$ gates between qubit pairs with 15 parameters per gate. Up to a global phase, $\mathrm{SU}(4)$ gates represent fully parametrized two-qubit interactions. By the KAK-decomposition [6], an $\mathrm{SU}(4)$ rotation can be decomposed into four single-qubit $\mathrm{U}(2)$ rotations (which are fully general single-qubit rotations), and the entangling gates, i.e., XX, YY, and ZZ. Concretely,

$$\begin{aligned} \mathrm{SU}(4)_{i,j}(\boldsymbol{\theta}) = {} & \mathrm{U}(2)_i(\theta_{1:3}) \times \mathrm{U}(2)_j(\theta_{4:6}) \times \\ & \mathrm{XX}_{i,j}(\theta_7) \times \mathrm{YY}_{i,j}(\theta_8) \times \mathrm{ZZ}_{i,j}(\theta_9) \times \\ & \mathrm{U}(2)_i(\theta_{10:12}) \times \mathrm{U}(2)_j(\theta_{13:15}), \end{aligned} \tag{1}$$

with a parameter vector $\boldsymbol{\theta}$ of length 15. The notation $\theta_{l:l+2}$ for $l = 1; 4; 9; 13$, refers to the three elements in the $\boldsymbol{\theta}$ vector that go into each $\mathrm{U}(2)$ gate, e.g., $\theta_{1:3} = [\theta_1, \theta_2, \theta_3]$. We note that PQCs with only $\mathrm{SU}(4)$ gates are redundantly parametrized because of the application of consecutive $\mathrm{U}(2)$ gates to the same qubit. When implementing on hardware, one may first compile the circuit into hardware-native gate sets and then combine trivially redundant gates.

In the case of the MPS initialized PQCs, the decomposed MPS tensors map to a $\mathrm{U}(4)$ rotation, which includes a global phase $e^{-i\phi}$ with $\phi \in [0, 2\pi]$. The $\mathrm{U}(4)$ were then decomposed into a trainable $\mathrm{SU}(4)$ rotation with a fixed global phase via the

KAK-decomposition:

$$\mathrm{U}(4) \xrightarrow{\text{KAK}} \mathrm{SU}(4)(\boldsymbol{\theta}) \times e^{-i\phi} \tag{2}$$

The $\mathrm{SU}(4)$ gates in the MPS initialized simulations that were not part of the linear layers, i.e., the additional gates that were used to extend the PQC layer to an all-to-all topology, were initialized by sampling the parameters from a normal distribution $\mathcal{N}$ with zero mean $\mu = 0$ and a small standard deviation $\sigma = 0.01$. This was also done for all gates in the near-identity initializations in Fig. 2 of the main text. The only exceptions were the additional gates in the VQE simulation in Fig. 2 of the main text and the large-scale gradient simulations in Fig. 4 of the main text, where $\sigma = 0$ was chosen. The former was done because of the sensitivity of the energy loss at these small scales, whereas the latter was for computational feasibility of the gradient calculation.

C. PQC simulation

All PQCs in this work were simulated using the Qulacs [7] quantum circuit simulator through the interface provided by the ORQUESTRA® platform [8]. All results utilized exact statevector simulation of the quantum states $\psi_{\boldsymbol{\theta}}$ and the corresponding probabilities $q_{\boldsymbol{\theta}}(\mathbf{x})$.

D. PQC optimization

The parameters of the PQC models in the main text were optimized using a Python implementation of the CMA-ES [9, 10] optimizer through the interface provided by the ORQUESTRA® platform [8]. In the case of the QCBM training, the initial step size was chosen as $\sigma_{\text{cma}} = 10^{-2}$ in all depicted cases. For the VQE optimization, we found the energy error at the scales $10^{-1} - 10^{-2}$ to be very sensitive to changes in parametrization. Therefore, we chose $\sigma_{\text{cma}} = 10^{-2}$ for the random and near-identity initializations, and $\sigma_{\text{cma}} = 7.5 \cdot 10^{-3}, \sigma_{\text{cma}} = 5.0 \cdot 10^{-3}$, and $\sigma_{\text{cma}} = 2.5 \cdot 10^{-3}$ for the MPS initialized modes with $\chi = 2, \chi = 4$, and $\chi = 8$, respectively. The population sizes λ_{cma} were always chosen to be $\lambda_{\text{cma}} = 20$, meaning that each iteration in Fig. 3 of the main text corresponds to 20 quantum circuit simulations. In addition to a limit to the number of optimization steps, i.e., 10000, 15000, and 15000 for the PQCs in Fig. 2 of the main text (for Cardinality, BAS, and the Heisenberg model respectively), we also set a loss tolerance of $5 \cdot 10^{-4}$ which may stop the optimization if differences of loss values between steps stay below this threshold. This can be observed in the $\chi = 8$ example in Fig. 2c of the main text.

E. Gradients

The gradients with respect to the KL divergence loss were calculated using a finite-distance gradient estimator

$$\frac{\partial \mathcal{L}(\boldsymbol{\theta})}{\partial \theta_l} = \frac{\mathcal{L}(\boldsymbol{\theta} + \epsilon \cdot \theta_l) - \mathcal{L}(\boldsymbol{\theta} - \epsilon \cdot \theta_l)}{2\epsilon} \tag{3}$$

with $\epsilon = 10^{-8}$ and for parameter index $l = 8$ in Eq. (1).

Supplementary Note 5. MPS DECOMPOSITION

Our MPS decomposition protocol used throughout this work was proposed and demonstrated in Ref. [11], and combines the analytical decomposition technique in Ref. [12] with intertwined optimization steps of the created unitaries using TN techniques on classical computers. Concretely, for a target MPS wavefunction $|\psi_{\chi_{\max}}\rangle$, the goal of the decomposition protocol is to output a sequence of circuit layers, i.e., $\prod_{i=k}^{1} U^{(i)}$, where $U^{(i)}$ represent the two-qubit unitaries in the linear quantum circuit layer i, such that the fidelity

$$\begin{aligned} f\big(\{U^{(i)}\}_i\big) &= \big|\langle 0^{\otimes N}| \prod_{i=k}^{1} U^{(i)\dagger} |\psi_{\chi_{\max}}\rangle\big| \\ &= \big|\langle 0^{\otimes N}| U^{(k)\dagger} \cdots U^{(1)\dagger} |\psi_{\chi_{\max}}\rangle\big| \end{aligned} \tag{4}$$

is maximized. The number of layers $k \leq K$ is chosen to be less or equal than a predetermined circuit depth limit K, or until a target fidelity $\hat{f}$ is achieved via $f\big(\{U^{(i)}\}\big) \geq \hat{f}$. The layer indexing convention from k to 1 is intentional as the decomposition protocol creates circuit layers "from the MPS backwards", such that the newest layer is implemented first in the quantum circuit.

The decomposition protocol sequentially creates circuit layers $U^{(i)}$ via the truncation and disentangling technique described in Ref. [12]. All existing circuit layers $\prod_{i=k'}^{1} U^{(i)}$ for a given decomposition iteration $k^{'} < k$ are optimized via the constrained optimization algorithm described in Ref. [13]. That algorithm leverages the *singular value decomposition* (SVD) and the fact that, for any matrix $\mathcal{M}$ that is decomposed via SVD, i.e., $\mathcal{M} = \mathcal{U} \cdot \mathcal{S} \cdot \mathcal{V}$, the product $\mathcal{U} \cdot \mathcal{V}$ represents the unitary matrix that best approximates $\mathcal{M}$. Ref. [11] shows that this protocol is very effective at decomposing MPS into a low number k of unitary circuit layers, especially with a limited computational budget for the optimization steps. The precision of the decomposition, i.e., the fidelity $f\big(\{U^{(i)}\}_i\big)$ in Eq. (4), can be sequentially improved with additional circuit layers and optimization steps. Additionally, unlike the method proposed in Ref. [14], the protocol is not hindered by cumulative approximation error build-up when only a limited number of circuit layers are employed.

---

[1] Andrew Arrasmith, Marco Cerezo, Piotr Czarnik, Lukasz Cincio, and Patrick J Coles, "Effect of barren plateaus on gradient-free optimization," Quantum **5**, 558 (2021).

[2] Edward Grant, Leonard Wossnig, Mateusz Ostaszewski, and Marcello Benedetti, "An initialization strategy for addressing barren plateaus in parametrized quantum circuits," Quantum **3**, 214 (2019).

[3] Zhao-Yu Han, Jun Wang, Heng Fan, Lei Wang, and Pan Zhang, "Unsupervised generative modeling using matrix product states," PRX **8**, 031012 (2018).

[4] Steven R White, "Density matrix formulation for quantum renormalization groups," Physical review letters **69**, 2863 (1992).

[5] Matthew Fishman, Steven R. White, and E. Miles Stoudenmire, "The itensor software library for tensor network calculations," (2020).

[6] Robert R Tucci, "An introduction to Cartan's KAK decomposition for QC programmers," arXiv preprint arXiv:0507171 (2005).

[7] Yasunari Suzuki, Yoshiaki Kawase, Yuya Masumura, Yuria Hiraga, Masahiro Nakadai, Jiabao Chen, Ken M. Nakanishi, Kosuke Mitarai, Ryosuke Imai, Shiro Tamiya, Takahiro Yamamoto, Tennin Yan, Toru Kawakubo, Yuya O. Nakagawa, Yohei Ibe, Youyuan Zhang, Hirotsugu Yamashita, Hikaru Yoshimura, Akihiro Hayashi, and Keisuke Fujii, "Qulacs: a fast and versatile quantum circuit simulator for research purpose," Quantum **5**, 559 (2021).

[8] "ORQUESTRA®," `https://www.orquestra.io/`.

[9] Nikolaus Hansen and Andreas Ostermeier, "Adapting arbitrary normal mutation distributions in evolution strategies: The covariance matrix adaptation," in *Proceedings of IEEE international conference on evolutionary computation* (IEEE, 1996) pp. 312–317.

[10] Nikolaus Hansen, Youhei Akimoto, and Petr Baudis, "CMA-ES/pycma on Github," (2019).

[11] Manuel S Rudolph, Jing Chen, Jacob Miller, Atithi Acharya, and Alejandro Perdomo-Ortiz, "Decomposition of matrix product states into shallow quantum circuits," Quantum Science and Technology (2023).

[12] Shi-Ju Ran, "Encoding of matrix product states into quantum circuits of one-and two-qubit gates," Physical Review A **101**, 032310 (2020).

[13] Tomonori Shirakawa, Hiroshi Ueda, and Seiji Yunoki, "Automatic quantum circuit encoding of a given arbitrary quantum state," arXiv preprint arXiv:2112.14524 (2021).

[14] James Dborin, Fergus Barratt, Vinul Wimalaweera, Lewis Wright, and Andrew Green, "Matrix product state pre-training for quantum machine learning," Quantum Science and Technology (2022).
